# Species-specific roles of sulfolipid metabolism in acclimation of photosynthetic microbes to sulfur-starvation stress

**DOI:** 10.1371/journal.pone.0186154

**Published:** 2017-10-12

**Authors:** Norihiro Sato, Ryohei Kamimura, Kodai Kaneta, Misato Yoshikawa, Mikio Tsuzuki

**Affiliations:** 1 School of Life Sciences, Tokyo University of Pharmacy and Life Sciences, Hachioji, Tokyo, Japan; 2 JST, Chiyoda-ku, Tokyo, Japan; CEA-Saclay, FRANCE

## Abstract

Photosynthetic organisms utilize sulfate for the synthesis of sulfur-compounds including proteins and a sulfolipid, sulfoquinovosyl diacylglycerol. Upon ambient deficiency in sulfate, cells of a green alga, *Chlamydomonas reinhardtii*, degrade the chloroplast membrane sulfolipid to ensure an intracellular-sulfur source for necessary protein synthesis. Here, the effects of sulfate-starvation on the sulfolipid stability were investigated in another green alga, *Chlorella kessleri*, and two cyanobacteria, *Synechocystis* sp. PCC 6803 and *Synechococcus elongatus* PCC 7942. The results showed that sulfolipid degradation was induced only in *C*. *kessleri*, raising the possibility that this degradation ability was obtained not by cyanobacteria, but by eukaryotic algae during the evolution of photosynthetic organisms. Meanwhile, *Synechococcus* disruptants concerning *sqdB* and *sqdX* genes, which are involved in successive reactions in the sulfolipid synthesis pathway, were respectively characterized in cellular response to sulfate-starvation. Phycobilisome degradation intrinsic to *Synechococcus*, but not to *Synechocystis*, and cell growth under sulfate-starved conditions were repressed in the *sqdB* and *sqdX* disruptants, respectively, relative to in the wild type. Their distinct phenotypes, despite the common loss of the sulfolipid, inferred specific roles of *sqdB* and *sqdX*. This study demonstrated that sulfolipid metabolism might have been developed to enable species- or cyanobacterial-strain dependent processes for acclimation to sulfate-starvation.

## Introduction

Sulfoquinovosyl diacylglycerol (SQDG), which includes sulfoquinovose as a negatively charged head group, is conserved widely among oxygenic photosynthetic organisms (reviewed in e.g., [1]). This anionic lipid, which accounts for ca. 10–20 mole% of total cellular glycerolipids, contributes to construction of the membranes of chloroplasts in plants and their postulated ancestor, cyanobacteria, with predominant localization in their thylakoid membranes. The SQDG synthesis system in oxygenic photosynthetic organisms includes UDP-sulfoquinovose synthase (encoded by *SQD1* in plants or *sqdB* in cyanobacteria) and SQDG synthase (encoded by *SQD2* in plants or *sqdX* in cyanobacteria). These two enzymes catalyze successive reactions, i.e., the former combines sulfite and UDP-glucose for UDP-sulfoquinovose synthesis, and then the latter transfers the sulfoquinovose moiety of UDP-sulfoquinovose to diacylglycerol for SQDG synthesis. Interestingly, physiological characterization of SQDG-deficient mutants, including those disrupted in the above genes for SQDG synthesis, has thus far shown species-dependent roles of SQDG under normal growth conditions or even strain-dependent ones within species: e.g., SQDG is essential for cell growth in a cyanobacterium, *Synechocystis* sp. PCC 6803 (herein referred to as *Synechocystis*, [2]), but not in other oxygenic photoysnthetic organisms including cyanobacteria *Synechococcus elongatus* PCC 7942 (herein referred to as *Synechococcus*, [3]) and *Synechococcus* sp. PCC 7002 [4], a green alga, *Chlamydomonas reinhardtii* [5], and a seed plant, *Arabidopsis thaliana* [6]. Meanwhile, SQDG is required for the normal functionality of photosystem II in *Synechocystis* [2] and *Chlamydomonas reinhardtii* [7], but not in *Synechococcus* [3], *Synechococcus* sp. PCC 7002 [4] or *Arabidopsis thaliana* [6].

Photosynthetic microbes, which could account for about a half of global net primary production [8], generally face deficiency in phosphate, the preferred environmental phosphorus (P)-source (e.g., [9]). The SQDG content is elevated in photosynthetic microbes in response to deficiency in phosphate (e.g., [10]). The increase of SQDG is accompanied by a decrease in the content of another anionic lipid, phosphatidylglycerol (PG), whereby the summed content of these two anionic lipids is maintained at a certain level. The similar lipid-remodeling in response to phosphate-limitation occurs in seed plants like *Arabidopsis thaliana*, and also in anoxygenic photosynthetic and non-photosynthetic root nodule-forming bacteria that possess SQDG [11–13]. It therefore is generally accepted that SQDG substitutes for PG for proper functionality of membranes and reduction in P-quota (e.g., [1]). Consistent with this idea, *sqdB/SQD1*- or *SQD2*-disrupted mutants including those of *Synechococcus*, *C*. *reinhardtii*, and *A*. *thaliana*, which are devoid of SQDG that should otherwise be increased in quantity, were much more severely repressed in cell growth during acclimation to phosphate-limitation, relative to the wild types [3, 6, 14].

Sulfur (S) is one of macronutrients for photosynthetic organisms, similar to P, and is the essential component of proteins and sulfolipids. Distinct from phosphate, sulfate, the preferred S-source for photosynthetic microbes, however shows extremely uneven distribution in aquatic habitats (reviewed in [15]): although the oceans typically contain sulfate at around 29 mM, i.e., supra-optimal concentrations for photosynthetic microbes, freshwater environments were highly variable in sulfate concentration such that lake sulfate ranged from as low as 10 to 1000 μM, dependent on the level of industrialization around the surrounding regions. Industrial emission of SO_2_ into the atmosphere could elevate the sulfate content of freshwater environments, which have however been decreased with the enforcement of legislations that limit SO_2_ emission in North America and Europe [16–18]. Therefore, there might be a growing trend that photosynthetic microbes in freshwater face S-deficiency. S-starvation was experimentally found to affect SQDG metabolism in a freshwater microbe, *C*. *reinhardtii*. Upon sulfate-deficiency, SQDG is almost completely degraded, whereby a major intracellular S-source for protein synthesis is ensured at an early stage, accompanied by an increase in the PG content [19]. Simultaneously, SQDG synthesis activity is enhanced through up-regulation of *SQD1* mRNA, which seems to be responsible for maintenance of SQDG at 5% of the normal level as a molecular tool to structurally stabilize the photosystem I complex [20]. However, behavior of SQDG metabolism and its physiological significance have never been examined under S-starved conditions in any freshwater microbes other than *C*. *reinhardtii*.

In this study, effects of S-starvation on SQDG metabolism was investigated in freshwater microbes such as cyanobacteria, *Synechococcus* and *Synechocystis*, and a green alga, *Chlorella kessleri*, to gain more insight into the taxonomical distribution of SQDG degradation ability. Also, disruptants of *Synechococcus* as to the *sqdB* and *sqdX* genes (Δ*sqdB* and Δ*sqdX*, respectively; [21]) were characterized as to the behavior of cells for acclimation to S-starvation stress to determine the physiological significance of SQDG synthesis. Obtained results will be discussed from an evolutionary aspect for oxygenic photosynthetic organisms.

## Materials and methods

### Strains and growth conditions

The strains used were *C*. *kessleri* 11h [22], the wild types of *Synechocystis* and *Synechococcus*, and the two disruptants of *Synechococcus* as to the *sqdB* and *sqdX* genes [21, 23]. The culture media for normal growth were 5-fold diluted Gamborg’s B5 [24] and BG11 [23] for *C*. *kessleri* and each cyanobacterial strain, respectively. S-free medium was prepared by replacing sulfate with chloride, i.e., (NH_4_)_2_SO_4_, MgSO_4_·7H_2_O, ZnSO_4_, CuSO_4_·5H_2_O, FeSO_4_·7H_2_0, and MnSO_4_·H_2_0 with NH_4_Cl, MgCl_2_, ZnCl_2_, CuCl_2_·2H_2_O, FeCl_2_·4H_2_0, and MnCl_2_·6H_2_0, respectively, in 5-fold diluted Gamborg’s B5, and MgSO_4_·7H_2_O, ZnSO_4_, and CuSO_4_·5H_2_O with MgCl_2_, ZnCl_2_, and CuCl_2_·2H_2_O, respectively, in BG11. Cells were cultured in glass tubes with continuous fluorescent lamp illumination (24 μmol photons m^-2^s^-1^) at 30°C with aeration, and harvested at the indicated times for physiological and biochemical analyses.

When needed, *C*. *kessleri* or cyanobacterial cells were universally labeled with ^35^S by culturing in the above [^32^S]sulfate-replete medium in the presence of [^35^S]sulfate (231 Mbq·nmol^-1^, 3.7 Mbq·mL^-1^). The labeled cells were washed three times with the S-free medium, and then shifted to ^32^S-replete or S-free conditions for further growth.

### Determination of pigment and lipid contents

The contents of Chl *a* and *b* were measured in *C*. *kessleri*, whereas Chl *a* and PBS were measured in cyanobacterial strains, as we previously described [24, 25]. Total lipids were extracted from the cells and subjected to TLC for separation into individual lipid classes, as previously described [7]. Total lipids and each lipid class were used for the synthesis of fatty acid methyl esters with 5% anhydrous methanolic HCl, and analyzed by capillary GLC, as described previously [7].

### Measurement of radioactivity of [^35^S]SQDG

During culturing of ^35^S-labeled cells under ^32^S-replete or S-free conditions, a portion of the culture was used for measurement of the radioactivity as total radioactivity. Another portion was used for extraction of total lipids, and subsequent separation of SQDG on a TLC plate and measurement of its radioactivity in the silica gel that was scraped off. The radioactivity of the culture and SQDG were measured with a liquid scintillation counter (LSC-6100 ALOKA, Tokyo, Japan), as described previously [20]. The radioactivity of SQDG was estimated relative to the total radioactivity.

### Semi-quantitative reverse transcriptase (RT)-PCR

RT-PCR was performed for the *Synechococcus* WT for investigation of expression of *sqdB*, *sqdX*, and *sqdBX* operon at the transcript level, with the use of the *rnpB* gene as an internal control, as described previously [25]. The primer sets used were shown in supplementary S1 Table.

## Results

### Induction of SQDG degradation in a green alga, *C*. *kessleri*

**Fig 1:**
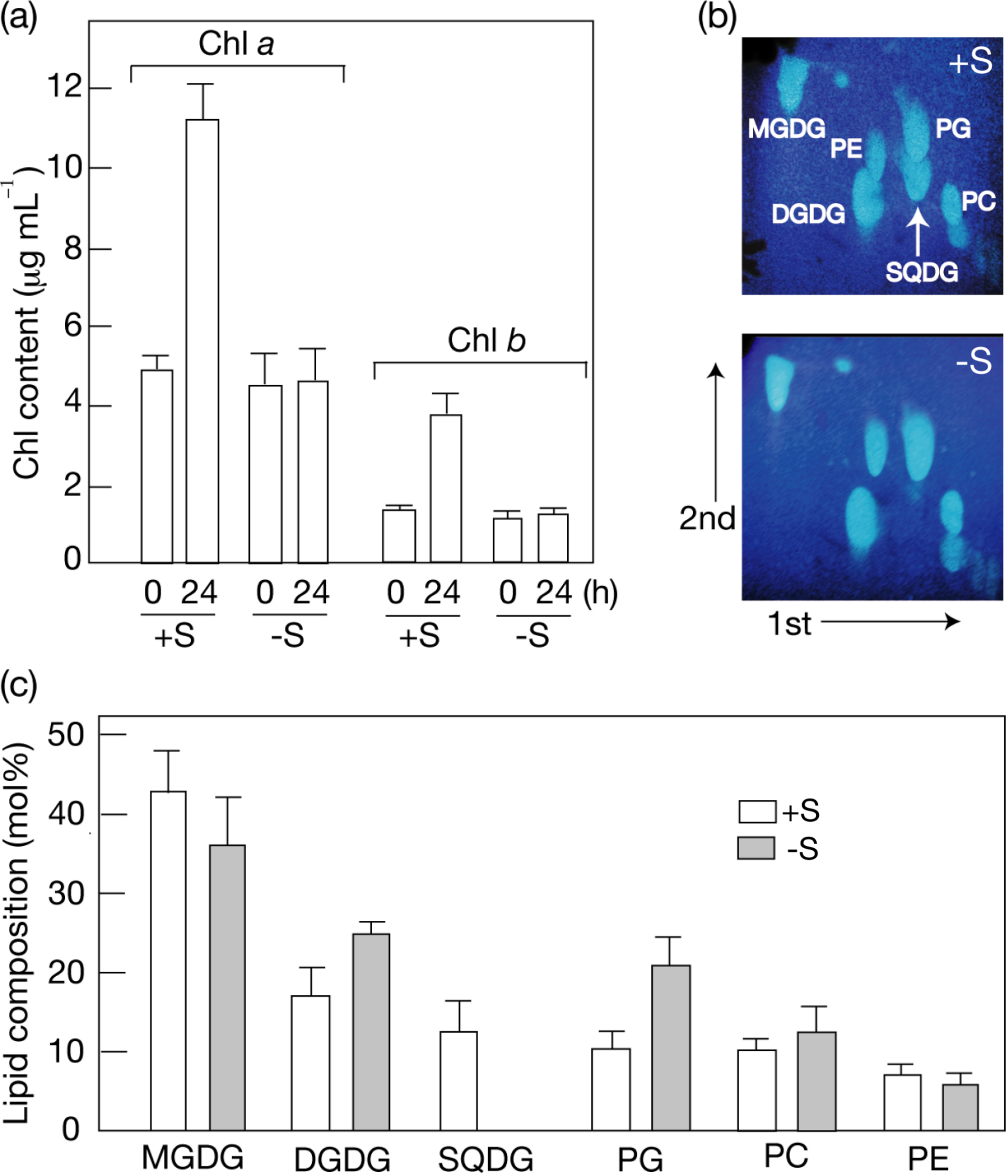
Effects of S-starvation on Chl and lipid contents in *C*. *kessleri*. (a) The respective contents of Chl *a* and *b* are shown after a shift of normally grown cells to S-replete (+S) or S-starved (-S) conditions for further growth for 1 day. (b) Profiles of polar lipids from +S and -S cells on two-dimensional TLC. The solvent systems used for development of total cellular lipids were CH_3_Cl/CH_3_OH/H_2_O (130:50:8 by vol.) and CH_3_Cl/CH_3_OH/conc. NH_3_ solution (130:70:10 by vol.) for the 1^st^ and 2^nd^ dimensions, respectively. (c) Polar lipid compositions of +S (open bars) and -S (grey bars) cells. MGDG, monogalactosyl diacylglycerol. DGDG, digalactosyl diacylglycefrol. PC, phosphatidylcholine. PE, phosphaidylethanolamine. The values shown are the averages ± SD for three distinct groups of data.

*C*. *reinhardtii* cells when starved of S exhibit almost complete degradation of preexisting SQDG within a day to ensure an internal S-source, which seems reasonable in view of dispensability of SQDG for its normal cell growth [5, 19]. Here, this SQDG-degradation ability was investigated in another green alga, *C*. *kessleri*. S-starvation caused *C*. *kessleri* cells to reveal typical symptoms [19], i.e., repression of Chl *a* and *b* accumulation (-S, cf., +S, Fig 1A), which probably reflected a delay in the synthesis of global proteins including photosystem complexes. S-replete cells of *C*. *kessleri* contained chloroplast lipids, monogalactosyl diacylglycerol (MGDG), digalactosyl diacylglycerol (DGDG), SQDG, and PG, and extrachloroplast lipids, phosphatidylcholine (PC) and phosphatidylethanolamine (PE) (Fig 1B and 1C; [22]). S-starved cells, however, lost SQDG, and concomitantly increased the PG content by 2-fold as if to quantitatively complement the SQDG-loss (Fig 1B and 1C).

**Fig 2:**
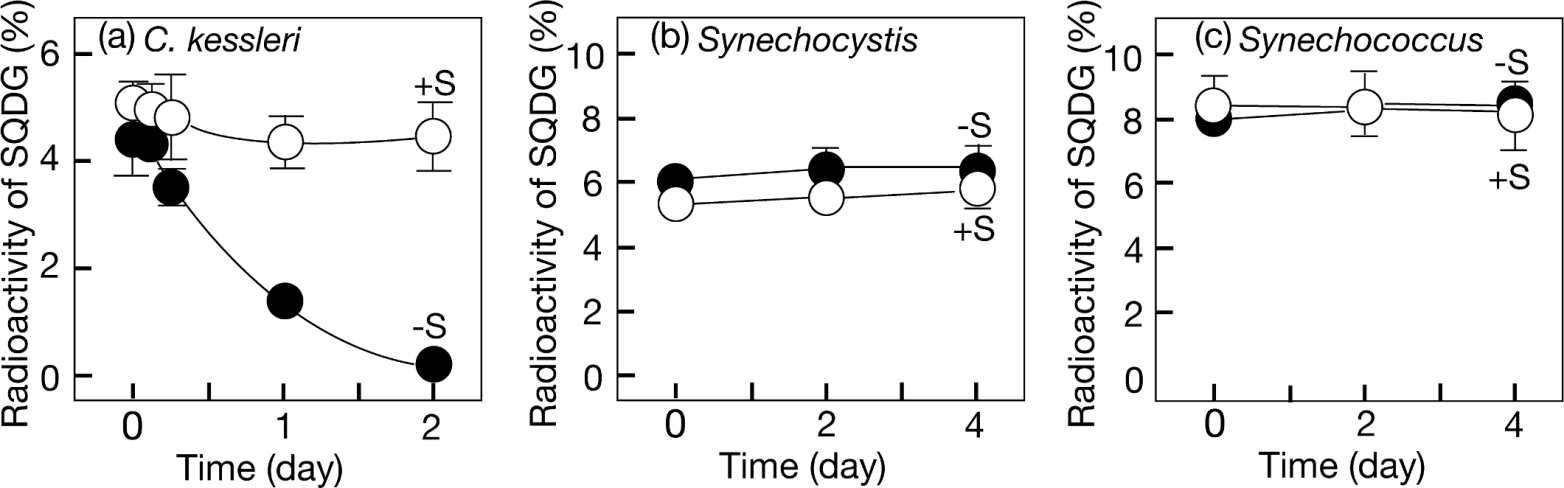
Effect of S-starvation on the stability of SQDG. Cells, which had been universally labeled with ^35^S, were transferred to non-label +S (^32^S, open circles) or -S conditions (closed circles), and thereafter the radioactivity of SQDG, relative to that in cells, was determined with time in *C*. *kessleri* (a), *Synechocystis* (b), and *Synechococcus* (c). The values shown are the averages ± SD for three distinct groups of data.

A radio-labeling experiment was then conducted to determine whether or not this defect in SQDG was due to positive degradation of it (Fig 2A). *C*. *kessleri* cells, which had been grown in the presence of [^35^S] sulfate for universal labeling, were shifted to non-label medium with or without [^32^S] sulfate, for further growth. The radioactivity of SQDG initially amounted to 5% of the total radioactivity in S-replete cells. S-replete conditions allowed the radioactivity of SQDG to be maintained at almost the same level during cell growth for 2 days, which indicated the high stability of SQDG. In contrast, the radioactivity gradually decreased under S-starved conditions, which proved that *C*. *kessleri* cells positively degrade SQDG in response to S-starvation.

### High stability of SQDG in cyanobacterial cells under S-starved conditions

**Fig 3:**
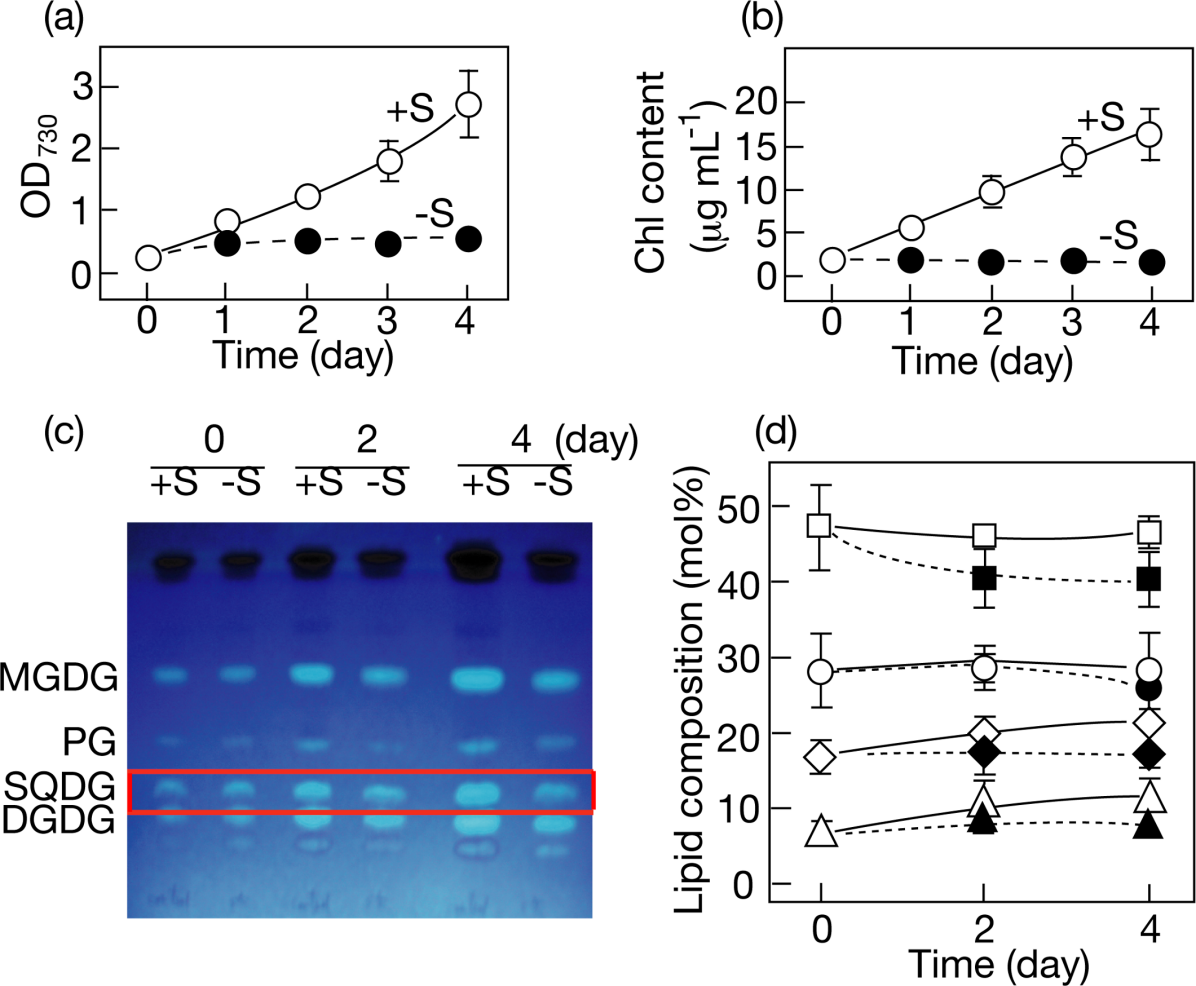
Effects of S-starvation on cell growth, and Chl and polar lipid contents in *Synechocystis*. OD_730_ (a) and the Chl *a* content (b) were determined for a culture of cells under +S (open circles) or–S (closed circles) conditions. (c) The changes in TLC profiles of polar lipids are shown for a culture of cells under +S or–S conditions. The solvent system used for development of total cellular lipids was CH_3_Cl/CH_3_OH/conc. NH_3_ solution (130:70:10 by vol.). (d) Changes in the polar lipid compositions are shown for cells grown under +S (open symbols) or–S (closed symbols) conditions. MGDG, squares. DGDG, diamonds. SQDG, circles. PG, triangles. The values shown are the averages ± SD for three distinct groups of data.

The effect of S-starvation on the SQDG content was then examined in *Synechocystis*, which requires SQDG for cell growth under normal conditions [2]. S-starvation caused markedly retarded growth of cells, Chl being maintained at the initial level throughout S-starvation for 4 days (Fig 3A and 3B). Concerning lipids, the most abundant lipid in S-replete cells was MGDG, followed by SQDG, DGDG, and PG in that order (Fig 3C and 3D; [23]). It was of note that S-starvation caused no drastic decrease in the SQDG content in *Synechocystis*. Labeling experiments with [^35^S] sulfate demonstrated that the radioactivity of SQDG initially corresponded to 6% of the total radioactivity in S-replete cells, and that the radioactivity of SQDG was maintained at this initial level irrespective of the presence or absence of an environmental S-source (Fig 2B).

**Fig 4:**
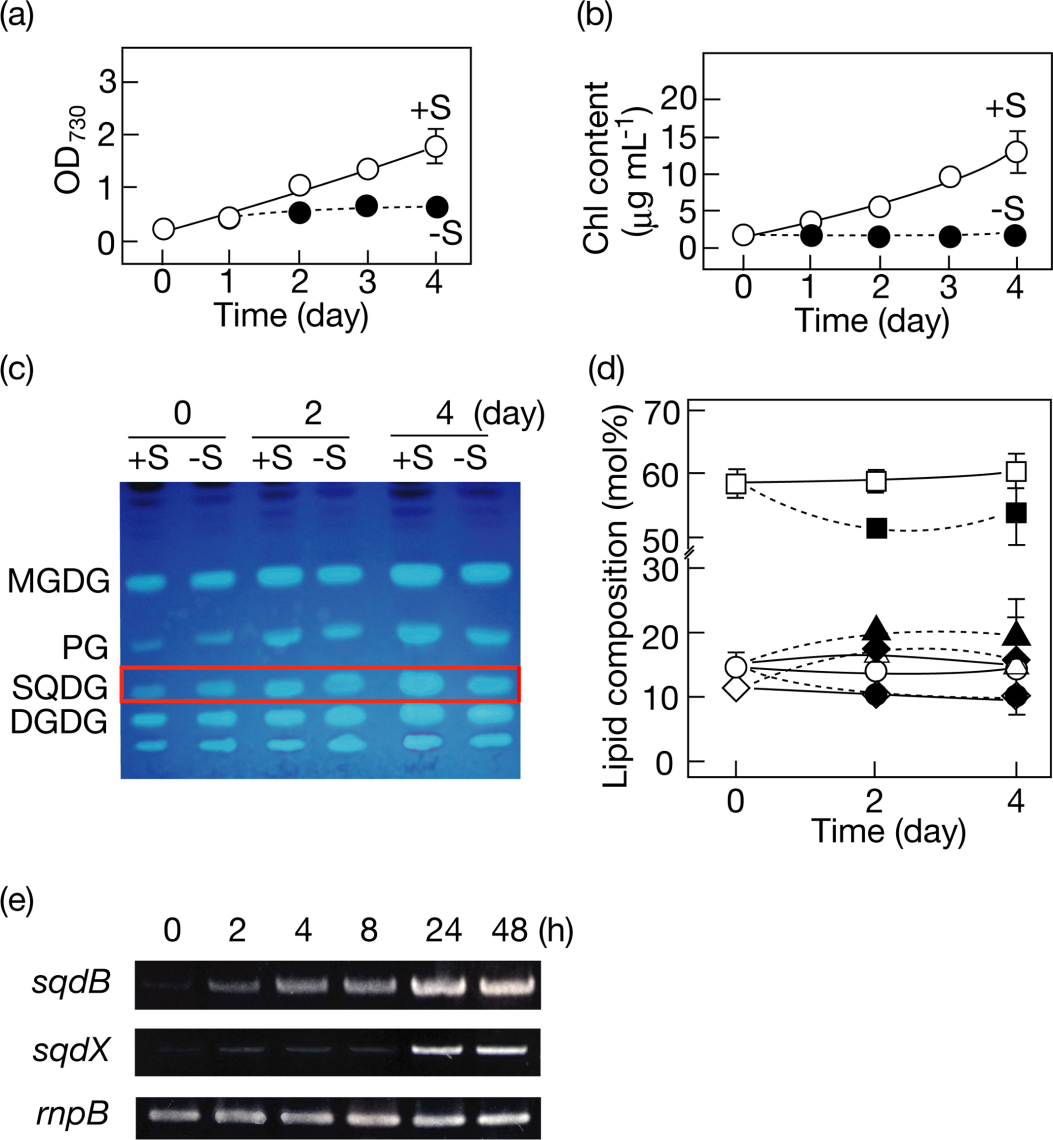
Effects of S-starvation on cell growth, Chl and polar lipid contents, and *sqdB*/*sqdX* genes in *Synechococcus*. OD_730_ (a) and the Chl *a* content (b) were determined for a culture of cells under +S (open circles) or–S (closed circles) conditions. (c) Changes in the TLC profiles of polar lipids are shown for a culture of cells under +S or–S conditions. The solvent system was the same as that in Fig 3C. (d) Changes in the polar lipid compositions are shown for cells grown under +S (open circles) or–S (closed circles) conditions. The symbols for lipids are the same as those in Fig 3D. (e) Expression of *sqdB*, *sqdX*, and *sqdBX* operon was examined in the WT under +S or–S conditions through RT-PCR. Expression of the *rnpB* gene for a subunit of ribonuclease P was investigated as an internal control. The values shown are the averages ± SD for three distinct groups of data.

*Synechococcus*, in which SQDG is dispensable for cell growth [3], also showed delayed cell growth in response to S-starvation, concomitantly with repressed Chl accumulation (Fig 4A and 4B). *Synechococcus*, similar to *Synechocystis*, exhibited no drastic decrease in the SQDG content (Fig 4C and 4D). Moreover, radio-labeling experiments demonstrated that the initial radioactivity of SQDG (8% of total one) was maintained at almost the same level throughout subsequent cell growth under S-starved conditions as well as under S-replete ones (Fig 2C). Collectively, SQDG is highly stable in cyanobacteria under S-starved conditions, irrespective of whether SQDG is essential or not.

### Physiological significance of the genes for SQDG synthesis in *Synechococcus* during acclimation to S-starvation

**Fig 5:**
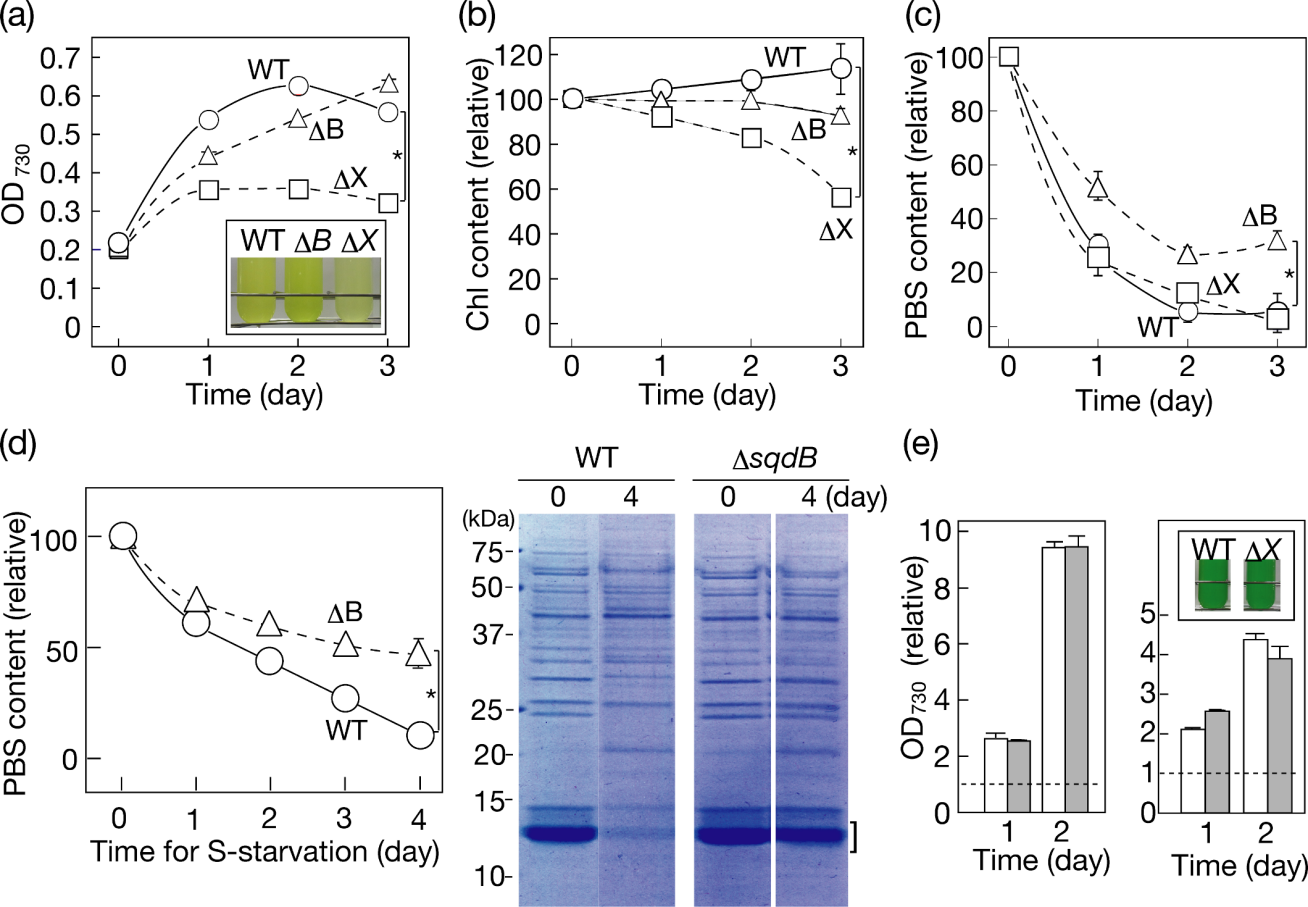
Effects of Δ*sqdB* and Δ*sqdX* in *Synechococcus* on cellular responses to S-starvation and on recovery of cell growth with re-supplementation of sulfate. Cell growth (a, OD_730_ values), and the Chl (b) and phycobilisome (c, d) contents during S-starvation were investigated in the WT (open circles), and disruptants of Δ*sqdB* (open triangles) and Δ*sqdX* (open squares), respectively. The inset in (a) shows photographs of normally, weakly, and strongly bleached cultures of the WT, and Δ*sqdB* (Δ*B*) and Δ*sqdX* (Δ*X*) disruptants, respectively. (d) Cells were starved for S with illumination with light at a milder intensity (14 μmol photons m^-2^s^-1^) than usual [(c), 24 μmol photons m^-2^s^-1^]. The phycobilisome content was determined by spectroscopy (c; d, left) or SDS-PAGE of soluble fractions of mechanically disrupted cells (d, right). (e) Cells starved of S for 3 days were shifted to S-replete conditions for recovery from the retarded growth. Left, S-starved cells of the WT (white bars) and Δ*sqdB* disruptant (grey bars) were initially adjusted to 0.1 in OD_730_ value with S-replete medium. Right, S-starved cells of the WT (white bars) and Δ*sqdX* disruptant (grey bars) were initially adjusted to an OD_730_ value of 0.2 with S-replete medium. The two-fold higher initial value of OD_730_ for the Δ*sqdX* disruptant relative to that for Δ*sqdB* one was to avoid possible photodamage in the Δ*sqdX* one, owing to its severe damages to cell growth and Chl accumulation (Fig 5A and 5B). Broken lines correspond to initial levels in the cultures. The inset in (e) shows photographs of 2-day cultures of the WT and Δ*sqdX* (Δ*X*). The values shown are the averages ± SD for three distinct groups of data. The significance of differences was evaluated by Student’s t test. *P < 0.05.

The Δ*sqdB* and Δ*sqdX* disruptants that we generated in *Synechococcus* retained *sqdX* and *sqdB* functionality, respectively, at some level [4], which would allow us to determine the independent roles, if any, of *sqdB* and *sqdX*, as well as the role of the final product, SQDG. Both disruptants, similar to the WT, showed relatively healthy growth under S-replete conditions such that the doubling time was 8.3 or 11.1h in Δ*sqdB* and Δ*sqdX*, respectively, c.f., 8.1h in the WT. Under S-starved conditions, the Δ*sqdB* disruptant showed retardation of cell growth and Chl accumulation in a manner similar to the WT (Fig 5A and 5B). However, phycobilisome (PBS) was retained in Δ*sqdB* at 30% of the initial level after 3-day starvation for S, in contrast to in the WT in which it was almost completely degraded (Fig 5C), as previously reported by Collier and Grossman [26]. The PBS degradation in WT markedly decreases the antenna size of photosynthesis to down-regulate photosynthetic electron transport activity, whereby the reducing potential of S-starved cells decreased to meet the low capacity of their cellular metabolism [27]. The PBS degradation in Δ*sqdB* was more seriously repressed such that >50% was retained with illumination with light at a milder intensity (Fig 5D, 14 μmol photons m^-2^s^-1^, cf., 24 μmol photons m^-2^s^-1^ in Fig 5C). Meanwhile, the Δ*sqdX* disruptant showed more pronouncedly delayed cell growth than the WT or the Δ*sqdB* one, concomitantly with an abnormal quantitative decrease in Chl, i.e., that in the PSI and/or PSII complexes (Fig 5A and 5B). However, PBS degradation proceeded normally in the Δ*sqdX* disruptant as in the WT (Fig 5C). Overall, it was found that the Δ*sqdB* and Δ*sqdX* disruptants, despite their common lack of SQDG, were disturbed in the physiological response in quite distinct manners. We also found that the expression level of *sqdB* or *sqdX* was markedly up-regulated in the WT during S-starvation through RT-PCR, especially 24 and 48 h after the onset of S-starvation (Fig 4E).

The WT, Δ*sqdB* or Δ*sqdX* cells starved of S were then cultured in the normal medium for investigation of recovery growth (Fig 5E). Despite defects in normal regulation of photosynthetic components and/or a concomitant defect in acclimating cell growth, Δ*sqdB* and even Δ*sqdX* cells (Fig 5E, right) exhibited normal recovery of cell growth with pigmentation. S-starved cells of *Synechococcus* thus seemed to show growth recovery independently of the system of SQDG synthesis, and its final product, SQDG.

## Discussion

### Evolutionary consideration of SQDG degradation under S-starvation

SQDG included S that accounted for 5–8% of the total cellular S in *C*. *kessleri*, *Synechocystis*, and *Synechococcus*, and was found to be stabilized in these respective species under S-replete conditions (Fig 2). These observations were compatible with the previous one for *C*. *reinhardtii* [19]. Through characterization of physiological defects in their SQDG-deficient mutants [1, 4], the physiological roles of SQDG in photosynthesis and/or cell growth has been found to depend on microbial species. We consider that SQDG is bound to the PSII core complex for the structural and functional integrity of the complex in *Synechocystis* and *C*. *reinhardtii* [2, 7]. In *Synechocystis*, the high stability of SQDG would therefore be useful for maintenance of the integrity of PSII. It will be a future subject to specify SQDG-dependent component(s) for DNA synthesis for progression of the cell cycle in *Synechocystis* [28], and evaluate the significance of the high stability of SQDG. Meanwhile, it seems that the high stability of SQDG demands properly regulated SQDG synthesis in *Synechococcus*, since an increase of the SQDG content through over-expression of the *sqdBX* operon disturbed its cell size determination [29].

Under S-starved conditions, SQDG degradation was induced probably for insurance of an intracellular S-source in *C*. *kessleri* as well as in *C*. *reinhardtii* [19, 20]. In contrast, the stability of SQDG was maintained at a high level in S-depleted cells of either cyanobacterial strain (Fig 2). These lines of evidence would be consistent with the idea that the ability to induce SQDG degradation under S-starved conditions could have been obtained not by cyanobacteria, but by algae, in the course of the evolution of oxygenic photosynthetic organisms, and already by the phase of diversification of green algae.

### Physiological significance of SQDG synthesis in *Synechococcus* cells during acclimation to S-starvation stress

Our observations as to the high stability of SQDG under S-starved conditions in *Synechocystis* and *Synechococcus* might reflect some crucial role of SQDG in cyanobacteria. In *Synechococcus*, however, it was definitely not SQDG, but the respective genes for SQDG synthesis, i.e., the *sqdB* and *sqdX* genes, that are necessary for regulatory rearrangement of the photosynthetic apparatus and/or acclimating cell growth (Fig 5). These requirements of *sqdB* and *sqdX* would be compatible with the high levels of their expression in S-starved cells (Fig 4E). PBS degradation proceeds through the *nbl* pathway including signaling components like *NblR* under S-starved conditions [27]. The *sqdB* gene might be involved directly or indirectly in some process required for S-starvation induced PBS degradation, which, however, would be unnecessary for the ongoing cell growth during acclimation. Meanwhile, it is probable that the *sqdX* gene is required for some fundamental process of physiology for the ongoing cell growth during acclimation to S-starvation, and also for some mechanism that structurally stabilizes the PSI and/or PSII complexes. It thus seems that photosynthesis is affected in distinct manners between S-starved cells of *sqdB* and *sqdX*. In an anoxygenic photosynthetic bacterium, *Rhodobacter sphaeroides*, a mutant impaired in the *sqdD* gene for SQDG synthase, which is structurally unrelated to that of oxygenic photosynthetic organisms, exhibited a much larger pool of UDP-sulfoquinovose than in the WT [30]. It will be a future project to investigate the molecular basis of how the distinct phenotypes of Δ*sqdB* and Δ*sqdX* cells were caused, including their distinctly disturbed patterns of photosynthetic parameters and those of SQDG metabolism.

Mutants impaired in lipid metabolism in cyanobacteria or chloroplasts of plants have thus far been examined as to their impact on the structural and functional integrity of the oxygenic photosynthetic apparatus, whereby, the correlation of lipids, i.e., the final products of the lipid synthesis pathways, to photosynthesis has been discussed (reviewed in, e.g., [31]). Such studies include those concerning SQDG- and/or PG-deficient mutants of *Synechocystis* or *C*. *reinhardtii*, which successfully verified the roles of these lipids in photosynthesis, through chemical complementation of the photosynthetic defects in the mutant by supplementation of the deficient lipid [2, 23, 32–34]. The causes of the defective phenotypes of photosynthesis that have so far been reported in other lipid mutants might have to be carefully re-evaluated, including possible accumulation of the substrates of enzymes encoded by the damaged genes.

In *Synechocystis*, its Δ*sqdB* cells, and probably Δ*sqdX* ones that have not been reported for its generation, have to be cultured in the presence of externally supplemented SQDG, owing to this cyanobacterial-strain specific essentiality of SQDG [2]. This particular feature becomes a hurdle to investigating the responsibility of *sqdB* and *sqdX* genes for acclimation to S-starved conditions. However, it is supposed that the role of *sqdB* under S-starved conditions in *Synechocystis*, if any, should exclude the responsibility for PBS degradation, since PBS is never subjected to positive degradation in S-starved cells of *Synechocystis* (supplementary S1 Fig, [35]). In other words, this study revealed a novel cyanobacterial-strain dependent role of SQDG synthesis or *sqdB* in *Synechococcus*, which might have been gained only by some group of cyanobacteria during their evolutionary diversification, or by cyanobacteria at an early phase of their evolution, followed by its persistent maintenance or loss during their evolutionary diversification. In either case, the role of *sqdB* in PBS degradation was not passed on to its counterpart, *SQD1*, in a green lineage where PBS was replaced completely by light-harvesting Chl-protein complexes [36]. At first, cyanobacteria could have integrated the SQDG synthesis system into essential physiological processes including acclimation to S-deficiency or DNA synthesis whereas green algae, after the evolutionary loss of such cyanobacterial roles of SQDG synthesis system, could have developed the system of SQDG degradation.

## Conclusions

This study demonstrated that freshwater photosynthetic microbes, which might face S-deficiency, utilize the SQDG metabolism for acclimation to S-deficiency in species or even cyanobacterial-strain dependent manner. This situation, which is reminiscent of the species-dependent physiological significance of SQDG under S-replete conditions, is quite distinct from the universal role of SQDG to substitute for PG under P-deficient conditions. Our findings will be a foundation for a fuller understanding of the species-dependent mechanisms by which photosynthetic microbes acclimate to S-deficiency stress, and for consideration of their changes through evolution of photosynthetic organisms. In line, with our study as a starting point, it is expected that research on lipid role will change to include consideration of their dependency on the organism, and also on the respective genes for lipid synthesis in an organism.

## Supporting information

S1 Table Primer sets used for semi-quantitative PCR analysis of transcripts as to the genes for SQDG synthesis. (DOC)

S1 Fig SDS-PAGE of the soluble fraction of *Synechocystis* cells starved for S. Note that PBS subunits were not decreased in their abundance during S-starvation.
(TIFF)
