# Supplementary material for: Species-specific roles of sulfolipid metabolism in acclimation of photosynthetic microbes to sulfur-starvation stress
Source: PLoS One. 2017 Oct 12;12(10):e0186154. doi: 10.1371/journal.pone.0186154 (PMC5638391; doi:10.1371/journal.pone.0186154)
Supplement: S1 Table — (DOC) [file pone.0186154.s001.doc]

S1 Table. Primer sets used for semi-quantitative PCR analysis of transcripts as to the genes for SQDG synthesis

Gene name Forward Reverse

*sqdB* 5’-GATTCTTGTATTGGGTGGCG-3’ 5’-GTTTCAGGAGCGCTGAGAGC-3’

*sqdX* 5’-CTTTACCGAGACGTTCCTCC-3’ 5’-AAGGGTAAGCAACCATCTGC-3’

*rnpB* 5’-AAGTCCGGGCTCCCAAAAGA-3’ 5’-TCTCTTGTCTTGGGCCGAAT-3’
