# Supplementary figures and images for: Species-specific roles of sulfolipid metabolism in acclimation of photosynthetic microbes to sulfur-starvation stress
Source: PLoS One. 2017 Oct 12;12(10):e0186154. doi: 10.1371/journal.pone.0186154 (PMC5638391; doi:10.1371/journal.pone.0186154)

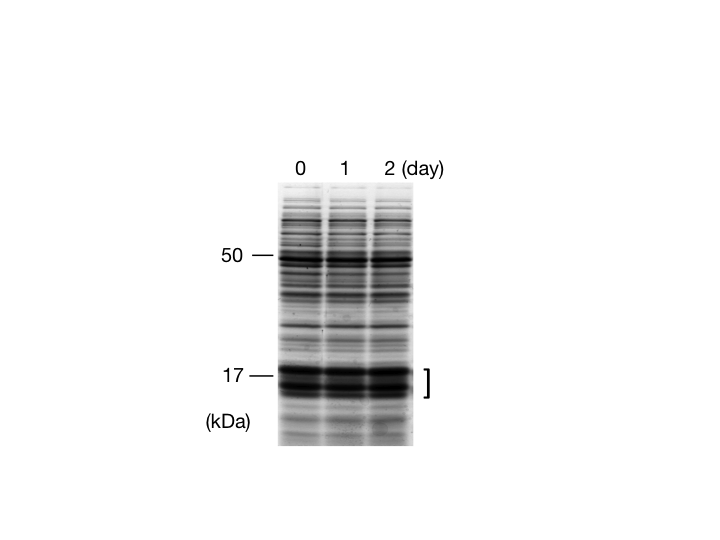

Supplement: S1 Fig — Note that PBS subunits were not decreased in their abundance during S-starvation. (TIFF) [file pone.0186154.s002.tiff]
